# Multi-trait genome-wide association analyses leveraging alcohol use disorder findings identify novel loci for smoking behaviors in the Million Veteran Program

**DOI:** 10.1038/s41398-023-02409-2

**Published:** 2023-05-05

**Authors:** Youshu Cheng, Cecilia Dao, Hang Zhou, Boyang Li, Rachel L. Kember, Sylvanus Toikumo, Hongyu Zhao, Joel Gelernter, Henry R. Kranzler, Amy C. Justice, Ke Xu

**Affiliations:** 1 Yale School of Public Health, New Haven, CT 06511 USA; 2 VA Connecticut Healthcare System, West Haven, CT 06516 USA; 3 Yale School of Medicine, New Haven, CT 06511 USA; 4 University of Pennsylvania Perelman School of Medicine, Philadelphia, PA 19104 USA; 5 Crescenz Veterans Affairs Medical Center, Philadelphia, PA 19104 USA

**Keywords:** Genetics, Psychology

## Abstract

Smoking behaviors and alcohol use disorder (AUD), both moderately heritable traits, commonly co-occur in the general population. Single-trait genome-wide association studies (GWAS) have identified multiple loci for smoking and AUD. However, GWASs that have aimed to identify loci contributing to co-occurring smoking and AUD have used small samples and thus have not been highly informative. Applying multi-trait analysis of GWASs (MTAG), we conducted a joint GWAS of smoking and AUD with data from the Million Veteran Program (*N* = 318,694). By leveraging GWAS summary statistics for AUD, MTAG identified 21 genome-wide significant (GWS) loci associated with smoking initiation and 17 loci associated with smoking cessation compared to 16 and 8 loci, respectively, identified by single-trait GWAS. The novel loci for smoking behaviors identified by MTAG included those previously associated with psychiatric or substance use traits. Colocalization analysis identified 10 loci shared by AUD and smoking status traits, all of which achieved GWS in MTAG, including variants on *SIX3, NCAM1*, and near *DRD2*. Functional annotation of the MTAG variants highlighted biologically important regions on *ZBTB20, DRD2, PPP6C*, and *GCKR* that contribute to smoking behaviors. In contrast, MTAG of smoking behaviors and alcohol consumption (AC) did not enhance discovery compared with single-trait GWAS for smoking behaviors. We conclude that using MTAG to augment the power of GWAS enables the identification of novel genetic variants for commonly co-occuring phenotypes, providing new insights into their pleiotropic effects on smoking behavior and AUD.

## Introduction

Smoking and alcohol use disorder (AUD) commonly co-occur in the general population [1]. Compared to the use of a single substance, smoking co-occuring with AUD has greater adverse health effects [2]. Smoking-related behaviors (e.g., smoking initiation, smoking cessation) and alcohol-related behaviors (e.g., alcohol consumption (AC) and AUD) have an estimated heritability of 40–50% [3–5]. The genetic correlations between smoking-related and alcohol-related behaviors are estimated to be about 40% [6, 7], suggesting that the pleiotropic effects of genetic variants contribute to their co-occurrence.

Genome-wide association studies (GWAS) with large sample sizes have made remarkable progress in identifying genetic loci for individual smoking-related and alcohol-related phenotypes. In a sample of over 1.2 million individuals, Liu et al. reported over 400 genome-wide significant (GWS) loci associated with multiple smoking-related and alcohol-related behaviors: 378 variants for smoking initiation, 24 variants for smoking cessation, and 99 variants for the number of alcoholic drinks consumed per week [8]. Quach et al. identified five loci for nicotine dependence in a meta-GWAS that included individuals with European ancestry and African ancestry [9]. In a sample of 209,915 European Americans (EA) from the Million Veteran Program (MVP), we reported 18 GWS loci for a smoking trajectory contrasting current versus never smoking (contrast I), which corresponds to smoking initiation, and five loci for another smoking trajectory contrasting current versus mixed smoking (contrast II), which is similar to smoking cessation [10]. Several dozen single nucleotide polymorphisms (SNPs) have been linked to alcohol misuse, AC, and AUD. For example, in another MVP study, we identified 13 loci for AC and 10 loci for AUD in an EA population [7]. In that study, as in others [11], AC and AUD were shown to have distinct genetic architectures.

Consistent with the phenotypic correlation between smoking- and alcohol-related behaviors, phenotypes related to these two substance use behaviors have moderate to strong genetic correlations [12, 13] and these correlations remain significant even after adjustment for environmental factors such as socioeconomic status [14]. However, the loci that contribute to the combined risks of smoking and drinking remain unclear, as standard GWAS considers traits in isolation rather than the combined influence of genetic variants on smoking and alcohol consumption or AUD. Thus, little is known regarding the pleiotropic effects of genetic variants on co-occuring smoking- and alcohol-related phenotypes.

A recently developed method, multi-trait analysis of GWASs (MTAG), enables joint analysis of genetically correlated traits to boost statistical power to detect variants for each trait [15]. MTAG takes summary statistics from single trait GWASs as input, generalizes inverse-variance-weighted meta-analysis to explore multiple traits, and calculates the trait-specific association for each variant. Moreover, MTAG accounts for overlap of samples among GWASs for different traits based on regression of linkage disequilibrium (LD) scores. Because of these features, MTAG has recently been applied to identify genetic variants for multiple, related phenotypes in psychiatric disorders and in substance use disorders. For example, Wu et al. utilized a sample size of approximately 60,000 EA individuals and identified genetic variants on seven genes commonly associated with four out of the following five psychiatric disorders: schizophrenia, bipolar disorder, autism spectrum disorder, attention-deficit hyperactivity disorder, and depression [16]. Recently, Deak et al. applied MTAG to discover novel risk loci for opioid use disorder [17], and Xu et al. applied MTAG for four common substance use disorders and reported several novel loci for opioid use disorder, cannabis use disorder, alcohol and smoking behaviors [18]. These studies show that MTAG is a useful method for identifying loci associated with strongly correlated psychiatric disorders, including substance use behaviors.

**Fig. 1:**
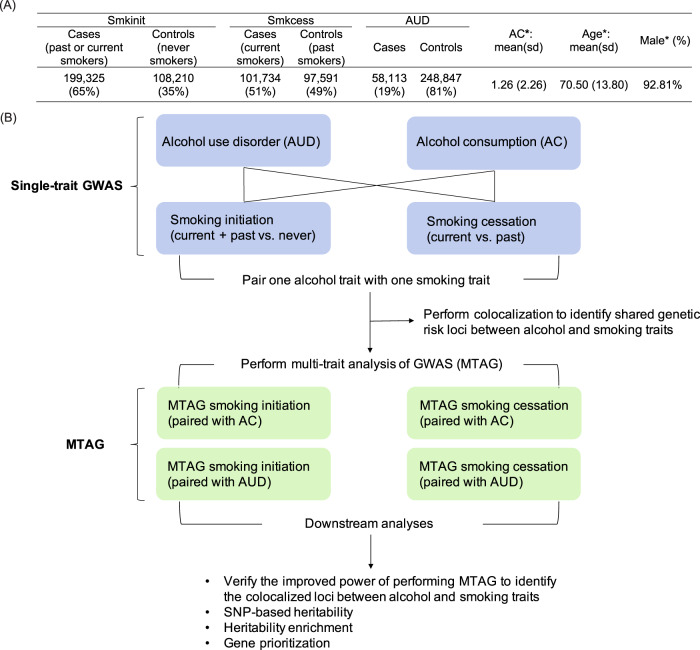
Phenotypes and analytic strategy. **A** The distribution of two smoking-related, two alcohol-related phenotypes, and demographic characteristics in the Million Veteran Program (MVP). **B** An overview of analyses performed on the single trait genome-wide association study (GWAS) and multi-trait GWAS (MTAG). All participants are from European American ancestry in the MVP (*N* = 318,694). *: the mean and standard deviation (sd) for AC and age, the proportion of male are calculated for the overall sample (*N* = 318,694). Smkinit: smoking initiation; Smkcess: smoking cessation; AUD: alcohol use disorder; AC: alcohol consumption measured by Alcohol Use Disorders Identification Test-consumption (AUDIT-C).

In this study, we performed MTAG for two smoking-related behaviors (smoking initiation and smoking cessation) and two alcohol traits (AC, defined the same way as Alcohol Use Disorders Identification Test–Consumption (AUDIT-C), and AUD) in 318,694 EA individuals from the MVP database. Single-trait GWAS was performed for each of the four phenotypes, deriving summary statistics for MTAG. Multi-trait colocalization served to identify genetic risk loci shared by smoking- and alcohol-related traits and to verify that MTAG augmented power to identify colocalized loci [19]. We also characterized MTAG performance by estimating the SNP-based heritability, heritability enrichment, and by prioritizing causal genes (Fig. 1). Our results provide novel insight for the genetic contribution to the co-occurance of smoking- and alcohol-related behaviors.

## Methods

### Study samples and phenotypes

The MVP recruited veteran volunteers and collected data from them using questionnaires, access to their electronic medical records (EMRs), and genomic analysis of blood samples. The Institutional Review Board (IRB) of the Veterans Affairs Central Office and site-specific IRBs approved the MVP study. All relevant ethical regulations for work with human subjects were followed in the conduct of the study, and informed consent was obtained from all participants prior to data collection.

We used flashpca to perform principal component analysis (PCA) on all MVP samples and 2504 samples from the 1000 Genomes Project (1KG) to identify the genetic ancestry of subjects [20], which was harmonized with self-reported race/ethnicity using the HARE (Harmonizing Genetic Ancestry and Self-identified Race/Ethnicity) method to construct ancestral groups [21]. We removed samples with a high genotype missing rate (>10%), discordant sex, excessive heterozygosity (>3 SD), and up to second-degree relatives. A total of 318,694 EAs, 81,057 African Americans (AA), and 31,828 Hispanic Americans (HA) passed quality control filters. In the analyses reported herein, we focused on the MVP EA samples. Among the MVP EA samples, the mean age was 70.50 years with a standard deviation of 13.80. Most participants were male (92.81%).

Using the available EMR smoking records, we identified 108,210 nonsmokers, 97,591 former smokers, and 101,734 current smokers. We defined smoking behaviors following Xu, Li, et al. [10]: individuals who report that they ever smoked (former or current smokers) were contrasted with those who report that they never smoked (nonsmokers) to study smoking initiation, while reported current smokers were contrasted with former smokers to explore smoking cessation.

For alcohol-related behaviors, we defined age-adjusted AUDIT-C (herein we call it AC) as described by Justice et al. [22], and the sample size for the quantitative phenotype AC was the overall sample size (*N* = 318,694). AUD cases were defined as individuals with ≥1 inpatient or ≥2 outpatient AUD codes according to the International Statistical Classification of Diseases and Related Health Problems, 9th (ICD-9) or 10th (ICD-10) revision; non-AUD (controls) were defined as the absence of any AUD code. The study sample comprised 58,113 individuals with AUD and 248,847 individuals without AUD.

### Genotyping and quality control

The MVP used an Affymetrix Axiom Biobank Array to genotype ~723,000 markers. SNPs were validated for common diseases and phenotypes of specific interest to the VA population (e.g., psychiatric traits) [23]. Minimac4 and the 1000 Genomes Project 3 reference panel were used to conduct genotype imputation [24, 25]. During the quality control step after imputation, we filtered out variants that were rare (minor allele frequency <0.01), had a missing rate >5%, an imputation *r*^2^ < 0.8, or that deviated significantly from Hardy–Weinberg equilibrium (*p* < 1E − 6). This yielded a total of 4.14 million variants. The genotype was based on Genome Reference Consortium Human Build 37 (GRCh37/hg19).

### Single trait GWAS for smoking and alcohol traits

For the smoking phenotypes (smoking initiation and smoking cessation) and AUD, logistic regression was applied to estimate marginal effects of each single genetic variant on the phenotype, while for AC, linear regression was used. PLINK (v1.9) was employed to perform logistic and linear regression analyses [26]. To be consistent with previous MVP GWAS on smoking traits [10] and on alcohol traits [7, 27], we adjusted the same covariates: age, sex, and the top 10 genotype principal components (PCs) calculated by flashpca [20].

### MTAG analysis for smoking-related behaviors

We used MTAG to jointly analyze summary statistics of one alcohol-related GWAS with one smoking-related GWAS [15], yielding a total of 4 combinations: AUD with smoking initiation, AUD with smoking cessation, AC with smoking initiation, AC with smoking cessation. The MTAG results for smoking behaviors were our focus. For each MTAG smoking trait, we further calculated ‘maxFDR’, which was an upper bound for the false-discovery rate (FDR) and was recommended to account for Type I error [15].

Lead SNPs and risk loci were defined in the same way for the single-trait GWAS and MTAG summary statistics: independent SNPs (LD, r^2^ < 0.1) with the most significant *p*-values were identified as lead SNPs, while the region containing all GWS variants (*p* < 5E − 8) that were in LD (*r*^2^ > 0.6) with the lead SNP was defined as a risk locus. ANNOVAR was then employed to map lead SNPs to their nearest genes [28], and loci within 250 kb were further merged into a single risk locus [10, 29, 30]. Each locus was represented by the top lead SNP with the minimum *p*-value [31].

### Colocalization between AUD and smoking-related behaviors

To identify genetic risk factors shared by AUD and smoking-related behaviors, we applied HyPrColoc (Hypothesis Prioritization for multi-trait Colocalization) [19] in multiple genomic regions using the full summary statistics from single-trait GWAS. HyPrColoc reports (1) the posterior probability that alcohol and smoking behaviors are colocalized in a specific region, (2) the causal variant in this colocalized region and the proportion of the posterior probability of colocalization explained by this variant. The identified shared risk variants facilitate validation of whether MTAG improves the power to identify colocalized loci between alcohol and smoking traits. We first used LDetect to partition the genome into 2258 independent regions (each, on average, approximately 1.6 cM in length) [32, 33], with LD estimated from the 1000 Genomes Project phase III samples of European ancestry [34], which is also the default reference panel in LDetect’s example [32]. We paired AUD with each of the smoking behaviors and performed colocalization analysis to identify shared genetic risk factors. For each pair, we reported the regions whose posterior probability of colocalization was greater than 0.75, as suggested by the authors of HyPrColoc [19]. LocusZoom (v1.3) was applied to visualize the change in regional associations after performing MTAG [35].

### Downstream analysis of the results from single-trait GWAS and MTAG

We estimated heritability and heritability enrichment for the 2 smoking-related single-trait GWASs and 2 smoking-related MTAGs. LD score regression (v1.0.0) was performed to estimate the narrow-sense heritability due to additive genetic effects [36]. To identify tissues most relevant to smoking-related phenotypes, we performed heritability enrichment analyses using 66 functional annotations from GenoSkyline-Plus (v1.0.0), which included tissues and cell lines from the blood, brain, lung, vascular system, heart, thymus, spleen, muscles, gastrointestinal tract, pancreas, liver, fat, bone/connective tissue, skin, breast, and ovary [37]. To adjust for multiple comparisons, we applied Bonferroni correction to the 66 enrichment tests for 2 smoking-related single-trait GWASs and 2 smoking-related MTAGs (*p* < 0.05/66/4 = 1.89E − 4).

Functional gene mapping was performed for 2 smoking-related MTAGs. We used the FUMA tool (v1.3.6) to conduct eQTL and chromatin interaction mapping [31]. We restricted eQTL mapping to 13 genotype-tissue expression (GTEx) v8 brain tissues and performed chromatin interaction mapping with the built-in adult cortex Hi–C data and enhancer/promoter annotations in 12 brain tissues from the Roadmap epigenomes. By default, we used false-discovery rate (FDR) < 0.05 for significant SNP-gene pairs in the eQTL mapping and FDR < 1E − 6 for significant chromatin interactions, as suggested by Schmitt et al. [38].

## Results

### Single-trait GWAS for smoking initiation, cessation, and alcohol behaviors

#### Single-trait GWAS for smoking initiation and cessation

We previously reported 12 GWS loci associated with smoking initiation and 8 loci associated with smoking cessation among 209,915 EA individuals in the MVP database [10]. In this study, where we utilized a larger sample from the MVP (*N* = 318,694), we identified 16 loci for smoking initiation (Supplementary Fig. 1, Supplementary Table 1), six of which were previously reported to be linked to smoking initiation (*LINC01360*, *TEX41, ZBTB20, EPHX2, NCAM1*, and *SPATS2*, Supplementary Fig. 2), including two identical lead SNPs, rs6438208 on *ZBTB20* and rs78875955 on *EPHX2*. In addition, we identified novel loci for smoking initiation, which included several RNA coding genes: *Y_RNA* and *LINC01833*. The intronic SNP rs4687552 on *ITIH3* was also a novel locus associated with smoking initiation in this sample.

We found eight GWS loci associated with smoking cessation, including three that we previously reported: rs6011779 on *CHRNA4*, rs17602038 near *DRD2* and rs11881918 near *CYP2A6* (Supplementary Fig. 1 and 2, Supplementary Table 1). We also previously reported a GWS association of rs112270518 near *DBH* with smoking trajectory II (current versus mixed smoking) [10], which corresponds to the smoking cessation trait. Here, rs3025360, near *DBH*, was a GWS locus associated with smoking cessation. Rs12341778 on *MAPKAP1*, with the mapped genes previously linked to mood disorder [39], also showed GWS associations with smoking cessation. The other two novel GWS loci associated with smoking cessation in the present study were 3:49638084:A:AAAATT on *BSN* and rs650599 on *SCAI*. Although the Manhattan plots for the 2 smoking traits stressed different loci and patterns, shared genetics is evident between these 2 traits (genetic correlation = 0.6642) (Supplementary Table 1).

#### Single-trait GWAS for alcohol phenotypes

Compared with our previous GWASs on AUD and AC in the MVP cohort [7, 27], here with a larger MVP sample size, we identified eight loci that overlapped with previously reported loci for AUD (i.e., *GCKR, SIX3, ARHGAP15, ADH1B, SLC39A8, CNTLN, DRD2*, and *FTO*) as well as six novel loci for AUD (*LNC01360, FANCL, LOC646736, PLCL2, KLB*, and *MTCH2*). Regarding AC, there were 27 GWS loci, six of which overlapped with loci associated with AUD, including five that we previously reported. Rs13130101, near *KLB*, was a GWS locus associated with AUD, and rs13146907, also near *KLB*, was a GWS locus associated with AC (Supplementary Table 1 and Supplementary Fig. 1, 2). *KLB* is a coreceptor for the hormone FGF21 and was previously linked to alcohol intake in a European ancestry population [40]. Altogether, we identified more loci for each smoking- and alcohol-related trait in the present study with a larger sample size drawn from the MVP database.

#### Genetic correlations between smoking- and alcohol-related phenotypes

The genetic correlation between smoking behaviors and AUD ranged from 0.59 to 0.62, substantially higher than the correlation between smoking behaviors and AC (ranging from 0.08 to 0.12) (Supplementary Fig. 3, Supplementary Table 1E). This pattern indicates that the shared genetic risk between smoking-related phenotypes and AUD is much greater than that between smoking-related phenotypes and AC.

Single-trait GWAS in AA and HA samples were summarized in Supplementary Figs 4 and 5. Few signals were detected, and they were not included in following analyses.

### MTAG analysis for smoking-related behaviors

We conducted a joint-GWAS for two smoking-related phenotypes (smoking initiation, smoking cessation) and two alcohol-related phenotypes (AUD and AC) using MTAG. Each smoking phenotype was paired with AUD or AC.

**Fig. 2:**
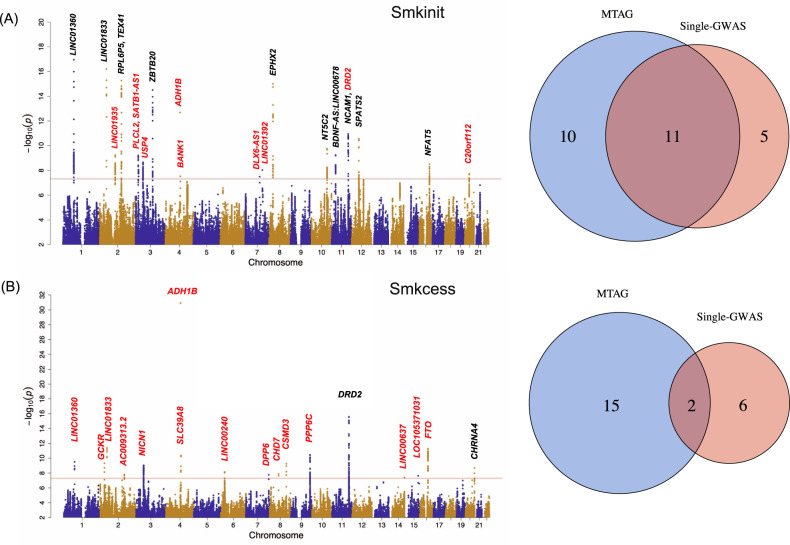
Multi-trait analysis of GWASs (MTAG) on two smoking phenotypes with alcohol use disorder (AUD). Manhattan plot of MTAG and Venn plot of the number of genome-wide significant (GWS) loci identified by single-trait GWAS and MTAG for **A** smoking initiation and **B** smoking cessation. The nearest genes to GWS loci are shown. New MTAG-identified loci are shown in red. Smkinit: smoking initiation; Smkcess: smoking cessation; AUD: alcohol use disorder.

Leveraging the summary statistics from single-trait GWAS for smoking initiation and AUD, MTAG identified 21 GWS loci for smoking initiation (maxFDR = 0.0021); five more than the number of loci identified through single-trait GWAS for smoking initiation. Among the 21 loci, 11 were identified by the single-trait GWAS for smoking initiation, while 10 were novel loci from the MTAG results (Fig. 2A). The novel loci identified by MTAG included the well-known loci for alcohol phenotypes, rs1229984 on *ADH1B* and rs6589386 on *DRD2* (Supplementary Table 2). Another novel locus for smoking initiation has been linked with smoking-related phenotypes in previous studies: rs6778080 on *USP4* was linked to the lifetime smoking index and depression [41, 42]. Of note, one locus identified in both single-trait GWAS and MTAG, rs4144892 on *NCAM1*, was also identified by a recent multivariate GWAS of externalizing liability at a nearby SNP rs9919558 [43]. Using MTAG, we were also able to replicate previous findings that linked genomic regions to smoking initiation in the Genetic Sequencing Consortium of Alcohol and Nicotine Use (GSCAN) study of 1.2 million individuals: [8] 9 loci identified in MTAG colocalized with GSCAN summary statistics (Supplementary Table 2).

Regarding smoking cessation, MTAG identified 17 GWS loci, 9 more than the single-trait GWAS for smoking cessation (maxFDR = 0.0103). Two of the 17 loci, rs6011779 on *CHRNA4* and rs17602038 on *DRD2*, were identified by both the single-trait GWAS and MTAG for smoking cessation. Fifteen of these loci attained GWS only in MTAG (Fig. 2B). As with the MTAG for smoking initiation, we found that multiple alcohol-related loci also attained GWS for smoking cessation. These loci included rs1229984 on *ADH1B* and rs62048402 on *FTO* (Supplementary Table 2). Another highly pleiotropic locus, rs13135092 on *SLC39A8*, previously associated with high-density lipoprotein cholesterol (HDL) in current drinkers [44] and schizophrenia [45], was a GWS locus associated with smoking cessation. Additionally, rs1260326 on *GCKR*, which has been linked to multiple metabolic traits [46], attained GWS for smoking cessation. Of note, 3 loci identified in MTAG colocalized with GSCAN summary statistics for smoking cessation:[8] *LINC00637, CHRNA4, PPP6C* (Supplementary Table 2).

The intersection of the MTAG loci, single-trait GWAS loci and previous reported MVP GWAS loci [10] was shown in Supplementary Fig. 6. The merging of loci within 250 kb (Methods) was shown in Supplementary Fig. 7.

Of note, compared with the single-trait GWAS results (Supplementary Fig. 1A, 1B), the MTAG of AC and smoking phenotypes did not yield new loci for smoking phenotypes (Supplementary Fig. 8). The subsequent analyses included only the results from the MTAG of each smoking trait and AUD. Together, these findings show that by leveraging the strongly correlated AUD trait, MTAG was able to identify more loci for smoking phenotypes than single-trait GWAS.

### Colocalization between AUD and smoking-related behaviors

**Fig. 3:**
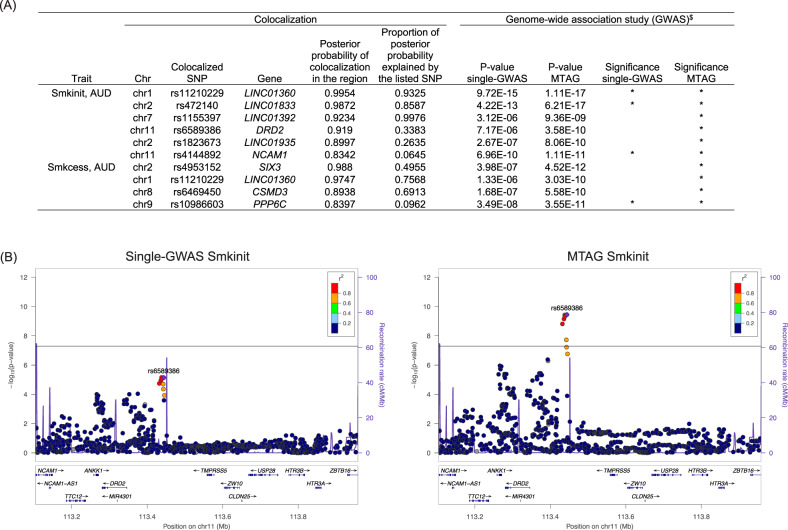
Multi-trait colocalization analysis of two smoking traits and alcohol use disorder (AUD). **A** Hypothesis prioritization for multi-trait colocalization (HyPrColoc) identified six regions shared by AUD and smoking initiation and four regions shared by AUD and smoking cessation. We report regions whose posterior probability of colocalization was greater than 0.75. ^$^: *p* values for the colocalized SNP in the single-trait GWAS and in MTAG for the corresponding smoking trait. **B** LocusZoom plots for the association of rs6589386 with smoking initiation. The genetic variant rs6589386 mapped near *DRD2* was identified as a colocalized SNP between AUD and smoking initiation. Smkinit: smoking initiation; Smkcess: smoking cessation; AUD: alcohol use disorder.

To identify genetic risk factors shared by AUD and smoking-related behaviors, we performed hypothesis prioritization for multi-trait colocalization (HyPrColoc) by pairing single-trait GWAS for each of the two smoking traits with single-trait GWAS for AUD. For each pair, we reported the regions whose posterior probability of colocalization was greater than 0.75 [19]. We identified a total of 10 colocalized regions, including six regions shared by AUD and smoking initiation and four shared by AUD and smoking cessation (Fig. 3A). Of note, among the 10 regions shared by AUD and smoking traits, MTAG identified all as attaining GWS, while single-trait GWAS identified only 4 out of 10 as GWS loci. Thus, the greater power of MTAG is most obvious for loci shared by AUD and smoking-related behaviors. For example, one colocalized SNP for AUD and smoking initiation, rs6589386 on *DRD2*, was marginally significant in the single-trait GWAS (*p*-value = 7.17E-06) but attained GWS in MTAG (*p*-value = 3.58E-10) (Fig. 3B).

Another noteworthy colocalized SNP shared by AUD and two smoking traits was rs11210229 on *LINC01360* (Supplementary Fig. 9). For smoking initiation, MTAG resulted in a moderate increase in the significance of the association with rs11210229 and other variants in LD. For smoking cessation, the increase in significance was greater: rs11210229 did not attain significance in the single-trait GWAS (*p*-value = 1.33E-06) but was the lead GWS SNP in MTAG (*p*-value = 3.03E-10).

### Estimated heritability and enrichment for smoking-related behaviors

The estimated heritability from MTAG was 1–2% greater for each smoking trait than the heritability from single-trait GWAS (Supplementary Table 3). For the two smoking traits, single-trait GWAS did not detect significant heritability enrichment, while MTAG identified significant heritability enrichment for smoking initiation in the anterior caudate (enrichment = 4.08, Wald test *p* = 1.86E-05) and the dorsolateral prefrontal cortex (enrichment = 5.41, Wald test *p* = 4.40E-05) as well as significant heritability enrichment for smoking cessation in the anterior caudate (enrichment = 5.64, Wald test *p* = 2.72E-06) and the colonic mucosa (enrichment = 7.69, Wald test *p* = 4.61E-05) (Supplementary Table 3).

### Prioritizing genetic regions for smoking phenotypes based on MTAG

**Fig. 4:**
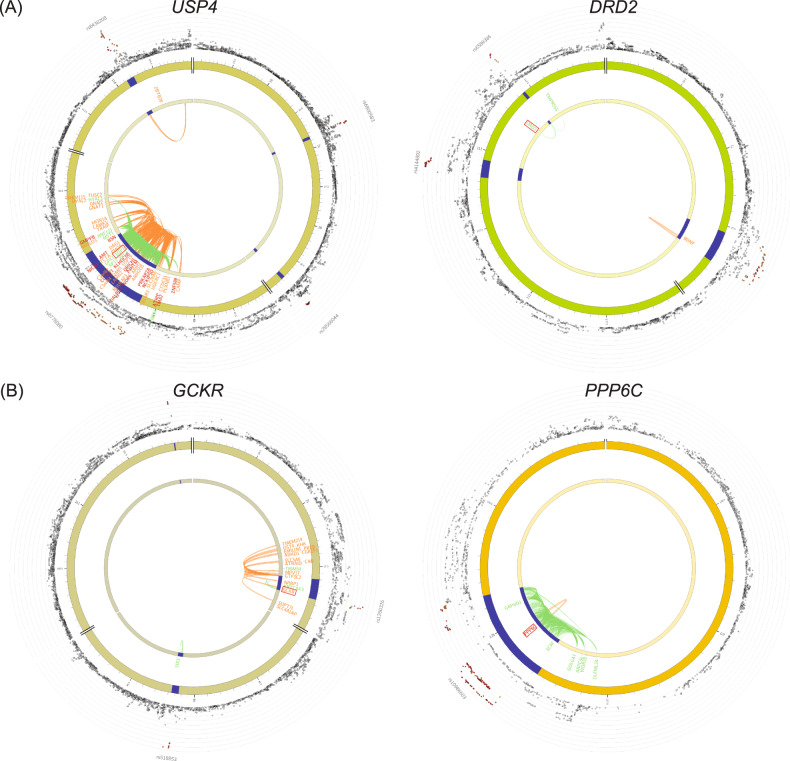
Gene prioritization for smoking traits using the MTAG. Functional mapping and annotation (FUMA) gene prioritization were performed for **A** smoking initiation and **B** smoking cessation. The outer layer shows chromosomal Manhattan plots. The GWS locus is indicated in blue. Genes mapped by chromatin interactions or eQTL are shown in orange or green, respectively. Genes mapped by both chromatin interactions and eQTL are shown in red.

By integrating MTAG variants with functional genomic features in brain tissues, we identified biologically important regions/genes for smoking behaviors. We performed expression quantitative trait loci (eQTL) and chromatin interaction mapping for the MTAG summary statistics of two smoking-related behaviors with the functional mapping and annotation (FUMA) tool [31]. For smoking initiation, we identified 41 genes mapped by eQTL and 85 genes mapped by chromatin interaction (Supplementary Table 4). Among those regions, seven overlapped with MTAG-identified loci, including two newly identified loci, rs6589386 on *DRD2* and rs6778080 on *USP4* (Fig. 4A); this finding indicates the pleiotropic effects of the MTAG-identified variants on gene expression and smoking behaviors. For smoking cessation, we identified 46 significant genomic regions by eQTL mapping and 67 regions by chromatin interaction mapping. Importantly, five of those regions overlapped with MTAG-identified loci, including four newly identified loci (Supplementary Table 4). For example, the MTAG-identified SNP rs10986603 on *PPP6C* colocalized with *PPP6C* eQTL in the anterior cingulate cortex and chromatin interaction in the cortex (Fig. 4B). *PPP6C* was recently associated with opioid addiction in EA individuals according to gene-based and eQTL analyses [47]. This finding was replicated in a GWAS on opioid use disorder conducted among MVP participants [48].

## Discussion

By leveraging the genetic architecture of AUD, we identified new loci for smoking behaviors that were not identified using a single-trait GWAS approach. MTAG revealed genetic variants that affect both smoking behaviors and AUD. Convergent evidence from MTAG, colocalization, and functional annotation analyses highlighted several AUD-associated genes that contribute to smoking behaviors. Importantly, the newly identified loci for smoking were colocalized with eQTL and chromatin interaction in brain regions previously shown to be relevant for addictive behavior. These findings underscore prior findings that MTAG is a powerful approach for identifying genetic variants for complex traits, as it is particularly important for revealing pleiotropic effects of significant variants that contribute to highly comorbid disorders. Although smoking and alcohol use are closely associated both epidemiologically and clinically, their genetic associations have not been well documented. Our study revealed GWS variants that likely contribute to the co-occurrence of these traits in a large EA population, thereby providing insight into their pleiotropic effects on the co-occuring phenotypes.

Performing colocalization between MTAG and GSCAN summary statistics [8], we replicated previous findings that linked genomic regions to smoking initiation and smoking cessation and identified some biologically meaningful overlaps. Among the 21 MTAG-identified loci for smoking initiation, nine colocalized with the GSCAN study, including three long intergenic nonprotein coding RNA genes (*LINC01360, LINC01833, LINC01392*). For smoking cessation, three of 17 MTAG-loci colocalized with the GSCAN study, including two novel loci (*LINC00637 and PPP6C*). However, AUD-associated genes such as *ADH1B* and *FTO* only attained GWS for smoking initiation or smoking cessation in the present study.

This study provides novel genomic findings that link well-established genetic loci for AUD to smoking behavior. These findings augment well-established phenotypic associations showing both high rates of smoking among individuals with AUD [49] and greater difficulty in stopping smoking among individuals with AUD [50]. A functional variant, rs1229984 on *ADH1B*, has long been recognized as a risk locus for AC and alcohol-related diseases across populations with different ancestry. Recently, this locus, combined with another functional locus on *ALDH2*, was shown to be predictive of smoking initiation in a Japanese population [51]. We found that rs1229984 was strongly associated with two smoking behaviors in the context of AUD, suggesting that rs1229984 influences smoking behavior for individuals with problematic alcohol use.

In addition, rs9937709, near *FTO*, was significantly associated with smoking cessation. *FTO* has been linked to obesity [52], AC, and AUD [7]. We previously reported that rs62033408, a lead SNP on *FTO*, was associated with AC and that rs1421085, near *FTO*, was associated with AUD. The MTAG results indicated that rs9937709, near *FTO* and 19.9 kb from rs1421085, was associated with smoking behaviors. Another region near *DRD2* was important for alcohol and smoking behaviors: we previously reported significant associations of rs61902812 with AUD and of rs3133388 with current versus never smoking, corresponding to smoking initiation. In this study, MTAG identified multiple novel loci near *DRD2* for different smoking behaviors, including rs6589386 for smoking initiation and rs17602038 for smoking cessation. Among them, a recent study on multivariate GWAS has reported rs6589386 on *DRD2* as the most significant signal associated with the addiction risk factor [53], a general genetic factor underlying problematic alcohol use, tobacco use, cannabis use disorder and opioid use disorder [54]. Altogether, these data suggest that this region is biologically important for understanding mechanisms underlying how variation in *DRD2* leads to addictive behaviors.

Despite smoking initiation and smoking cessation being very different behaviors, their genetic architectures are significantly correlated: in GSCAN, genetic correlation between initiation and cessation was 0.396 (se = 0.021) [8]. In our current study, the genetic correlation between two smoking traits was 0.6642 (se = 0.0401). One possible explanation for these findings is the complexity of smoking phenotypes [55]. Cigarette smoking has different stages such as initiation, experimentation, regular use, dependence, cessation, and relapse [56–58]. Smoking initiation is defined as “ever versus never” while cessation is defined as “current versus former”. Most studies used cross-sectional self-reported data. However, changes in individuals’ smoking status are not uncommon in the population. Future studies using long-lasting biomarkers to define never, former, and current smokers should enhance gene identification. Secondly, a proportion of genetic loci and pathways are shared between initiation and cessation traits. Wang and Li reported six shared pathways between initiation and cessation, including “Dopamine receptor signaling”, “calcium signaling”, “cAMP-mediated signaling”, “G-protein-coupled receptor signaling”, “Tyrosine metabolism”, and “Tight junction” [59]. Thus, shared loci for smoking traits and AUD from MTAG analysis are not surprising.

In contrast to the highly informative MTAG for AUD and smoking behaviors, the MTAG for AC and smoking behaviors did not show enhanced effects relative to the single-trait GWAS. Although the reasons for these differences are unclear, we hypothesize that AC and AUD may have different genetic architectures. As we reported, AC and AUD have distinct profiles of genetic correlations [7, 11]. AC is negatively genetically correlated with some medical diseases, such as coronary artery disease and Type 2 diabetes, while AUD is positively genetically correlated with psychiatric diseases, including some addictive disorders. In contrast, among all these psychiatric disorders, only major depressive disorder demonstrated a significant (negative) genetic correlation with AC [7]. This suggests that AUD reflects more information about addiction, and is thus more genetically correlated with smoking initiation and cessation. In the MTAG results presented here, the genetic correlations between AUD and smoking behaviors were approximately 0.6, while those between AC and smoking behaviors were approximately 0.1. This pattern is consistent with the original report [15], which emphasized that MTAG is most useful for analyzing phenotypes with strong genetic correlations. Another explanation is that the AC measurements may have been inaccurate. The AUDIT-C component of the MVP study is a self-reported measure of alcohol intake over the past 12 months. As we previously reported, individuals who were lifetime abstainers or former drinkers could have a confounding effect on gene associations with AC [60]. After removing former alcohol drinkers, single-trait GWAS identified more loci for AC in a sample from the UK Biobank. Future studies using longitudinal assessment of AC are warranted to deepen our understanding of the genetic architecture of smoking behaviors in the context of AUD and AC.

We acknowledge several limitations in the study. A lack of ancestral diversity limits the findings to EA individuals, due to lack of availability of GWASs on non-EA individuals that are large enough to provide adequate statistical power. As larger samples of other ancestral groups become available, we plan to examine whether the findings reported here are replicable in other populations. Second, MTAG is established on the assumption that all SNPs share the same variance–covariance matrix of effect sizes across multiple traits [15]. This assumption may not be applicable to AUD and smoking behavior. Authors of MTAG also stressed one potential problem for SNPs that are true null for one trait but non-null for another trait. For such SNPs, MTAG could have false positives in the first trait [15]. This statement is consistent with a recent study on opioid use disorder, which showed that increased detection of MTAG might come with a loss of specificity [17]. In our analyses, we quantified false discoveries by maxFDR (0.0021 for smoking initiation, and 0.0103 for smoking cessation), and low maxFDR values enhanced the confidence for the MTAG identified variants. Some of the newly identified loci by MTAG were shared between AUD and smoking behaviors by colocalization, such as rs6589386 on *DRD2*. However, there were also MTAG-identified loci that were not significant in the colocalization analysis, such as rs1229984 on *ADH1B*, suggesting that some loci identified by MTAG may be biased towards AUD. The MTAG-identified rs1229984 on *ADH1B* for smoking traits need to be interpreted with caution. Further investigations are needed to verify whether this locus revealed by MTAG is truly shared across different phenotypes. Recently, Xu et al. applied MTAG on four substance use traits, and reported that MTAG-derived polygenic risk scores (PRS) showed stronger associations with expected phenotypes than PRS derived from single-trait GWAS [18]. If a large independent cohort was available, comparison of predictive power of PRS constructed from single-trait GWAS versus MTAG association statistics might further support the utility of our MTAG findings. Third, we were unable to differentiate lifetime abstainers from former alcohol users who quit drinking (possibly due to alcohol-related problems) in the MVP samples. Future studies that use biomarkers instead of self-reported data to quantify AC could benefit from greater statistical power to detect risk loci for smoking behaviors. Finally, we did not examine the functional effects of the pleiotropic loci for smoking and AUD; such studies are needed to understand the mechanisms underlying our findings.

In summary, we identified multiple genetic loci significantly associated with the co-occurrence of smoking behavior and AUD in an EA population. The findings highlight several biologically relevant regions for further study that could elucidate potential mechanistic targets for therapeutic intervention shared by smoking behavior and AUD.

## Supplementary information

Supplementary Table 1

Supplementary Table 2

Supplementary Table 3

Supplementary Table 4

Supplementary Figures 1-9

## Data Availability

The full summary-level association data of the smoking-related traits from this report are available through dbGaP: (the accession number is phs001672.v1.p1). All codes for analysis are also available upon a request to the corresponding author.
