# Supplementary material for: Multi-trait genome-wide association analyses leveraging alcohol use disorder findings identify novel loci for smoking behaviors in the Million Veteran Program
Source: Transl Psychiatry. 2023 May 5;13:148. doi: 10.1038/s41398-023-02409-2 (PMC10162964; doi:10.1038/s41398-023-02409-2)
Supplement: Supplementary file 5 — Supplementary Figures 1-9 [file 41398_2023_2409_MOESM5_ESM.docx]

**
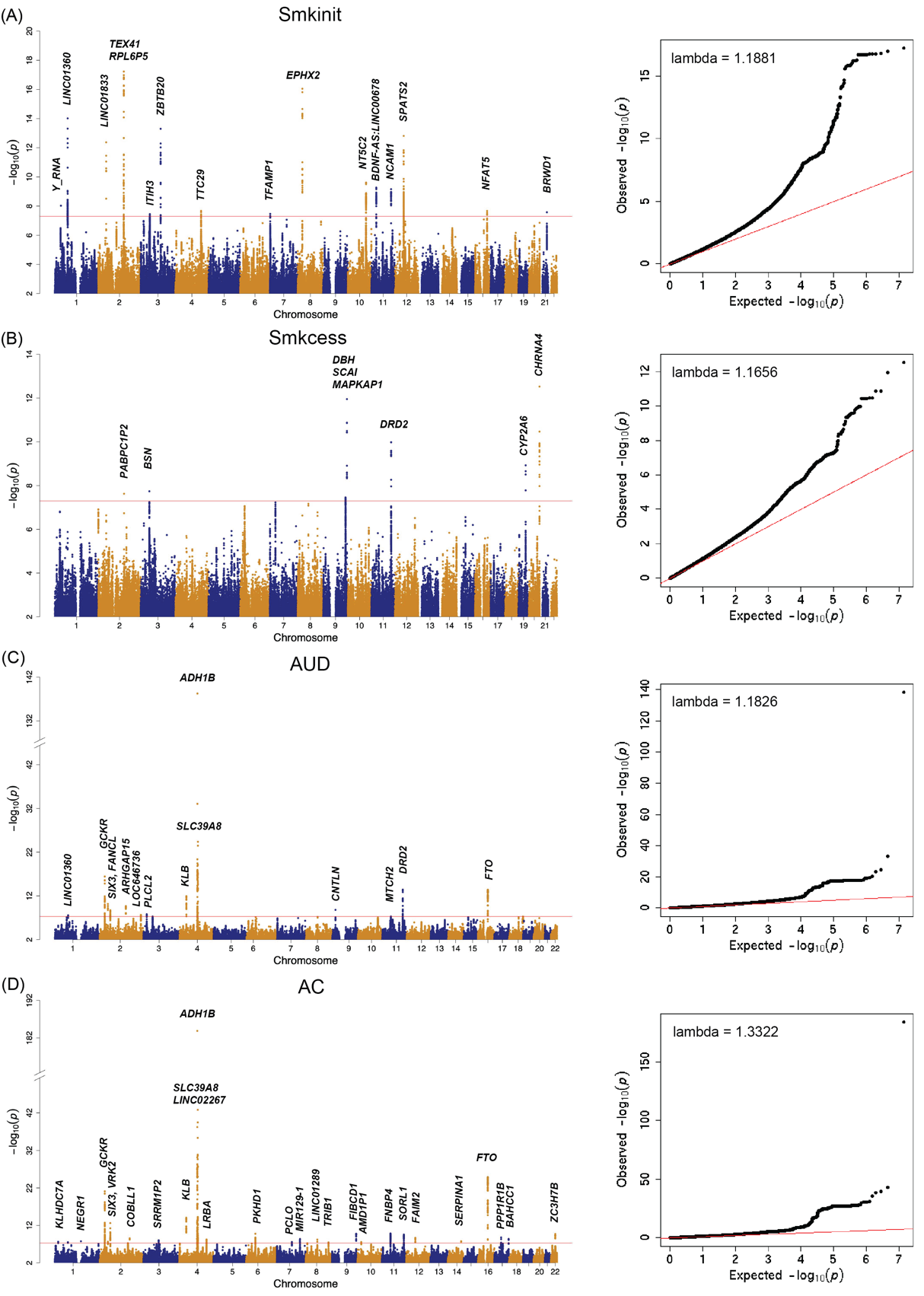
**

**Supplementary Figure 1: Single-trait genome-wide association study (GWAS) on smoking and alcohol phenotypes.** Manhattan plot and QQ plot of single-trait GWAS for (A) smoking initiation, (B) smoking cessation, (C) AUD, and (D) AC. The nearest genes for genome-wide significant (GWS) loci are shown.

Smkinit: smoking initiation; Smkcess: smoking cessation; AUD: alcohol use disorder; AC: alcohol consumption.


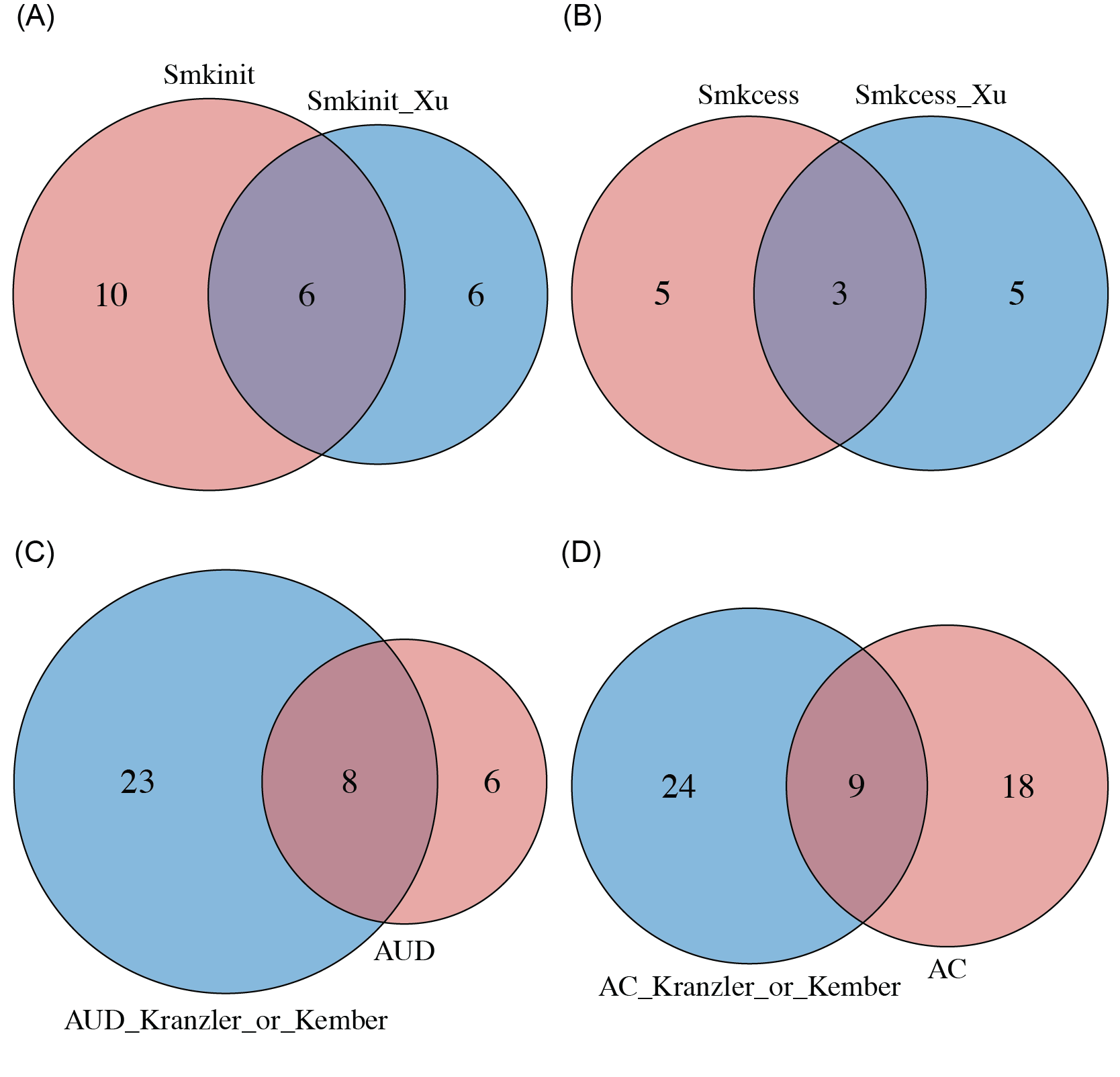


**Supplementary Figure 2: Venn plot of the number of genome-wide significant (GWS) loci identified by single-trait genome-wide association study (GWAS) and previous Million Veteran Program (MVP) GWAS.** Number of overlapped loci between single-trait GWAS and previous MVP GWAS for (A) smoking initiation, (B) smoking cessation, (C) AUD, and (D) AC.

Smkinit: smoking initiation; Smkcess: smoking cessation; Smkinit_Xu/Smkcess_Xu: previous MVP GWAS on smoking traits reported by Xu et al; AUD: alcohol use disorder; AC: alcohol consumption; AUD_Kranzler_or_Kember/AC_Kranzler_or_Kember: previous MVP GWAS on alcohol traits reported by Kranzler et al or Kember et al.


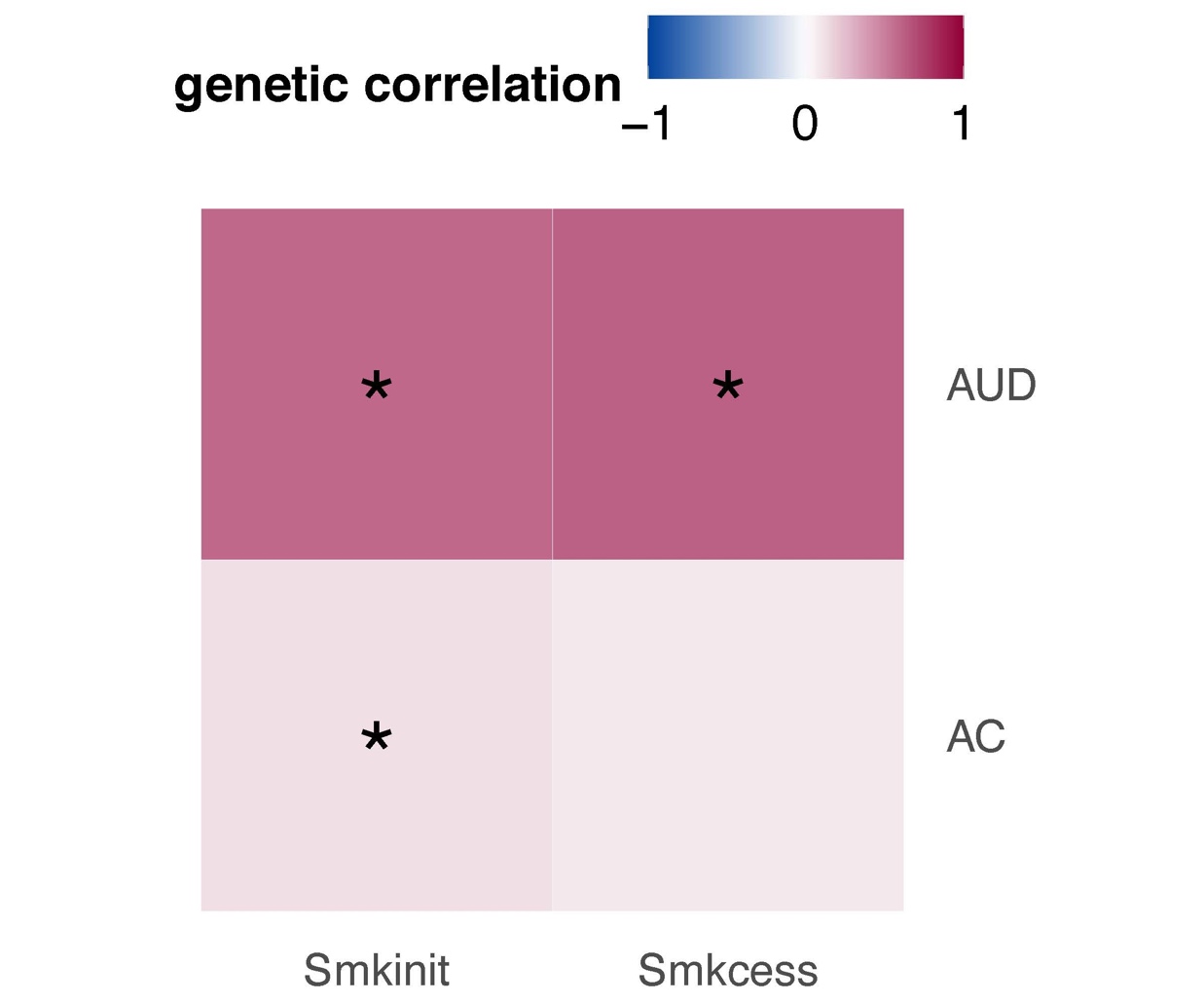


**Supplementary Figure 3: Genetic correlation between smoking and alcohol traits estimated from single-trait GWAS.** Genetic correlations of one smoking trait with one alcohol trait were estimated. The darkness of color indicates the magnitude of the genetic correlation, and the asterisk (*) indicates significance. The genetic correlation was considered significant if p < 0.05/4 = 0.0125 (Bonferroni correction).

Smkinit: smoking initiation; Smkcess: smoking cessation; AUD: alcohol use disorder; AC: alcohol consumption.

*
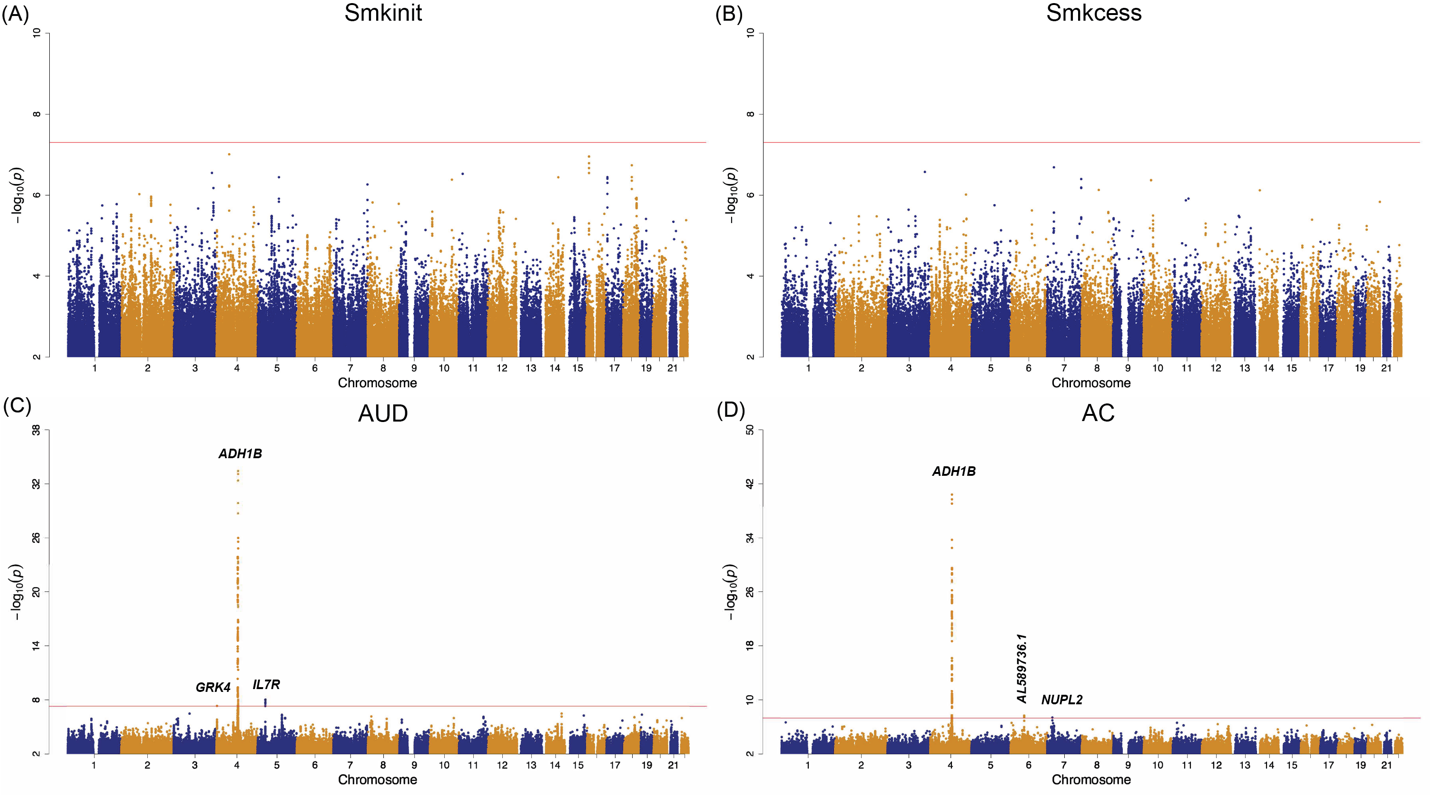
*

**Supplementary Figure 4: Single-trait genome-wide association study (GWAS) on smoking and alcohol phenotypes in 81,057 African Americans (AA).** Manhattan plot for (A) smoking initiation, (B) smoking cessation, (C) AUD, and (D) AC. The nearest genes for genome-wide significant (GWS) loci are shown.

Smkinit: smoking initiation; Smkcess: smoking cessation; AUD: alcohol use disorder; AC: alcohol consumption.

*
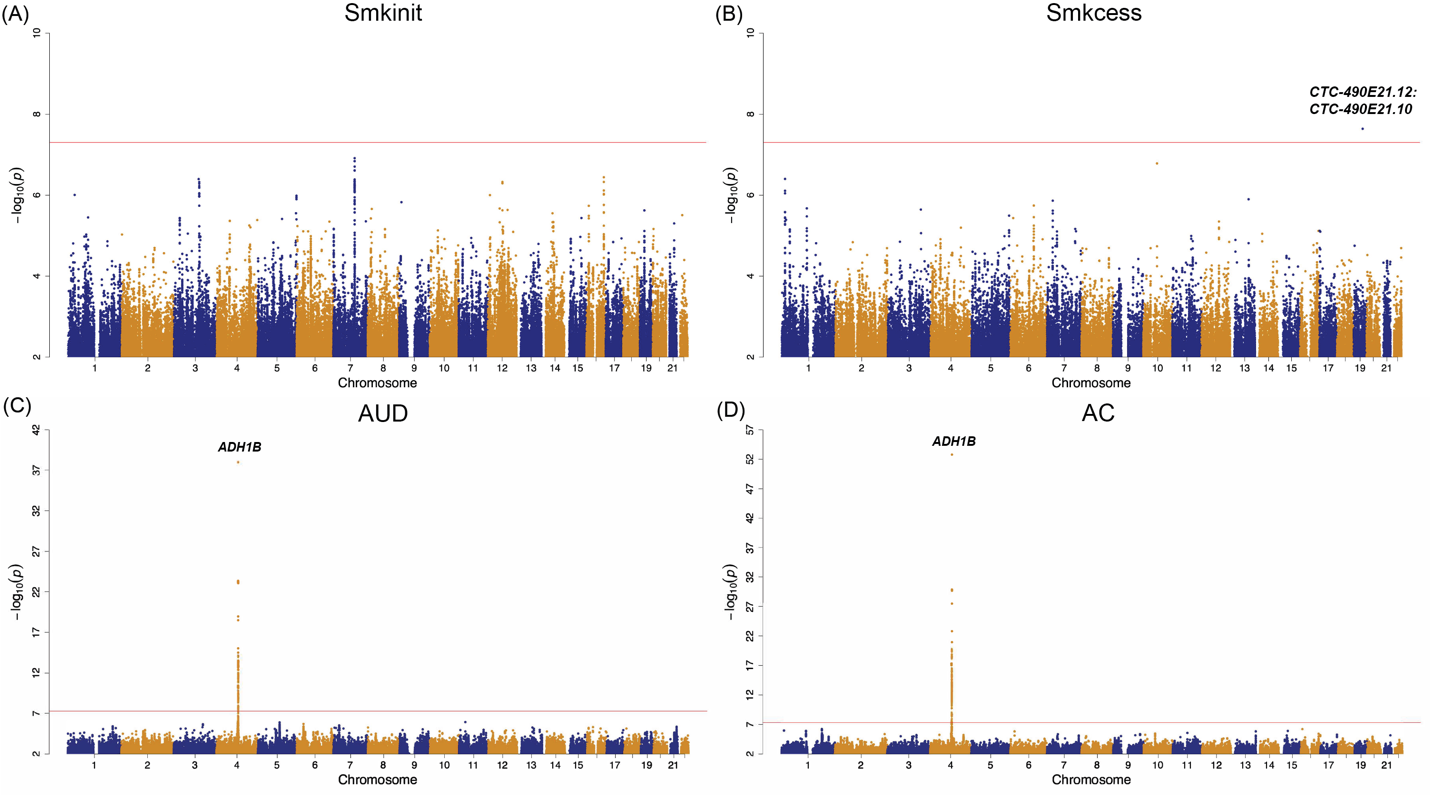
*

**Supplementary Figure 5: Single-trait genome-wide association study (GWAS) on smoking and alcohol phenotypes in 31,828 Hispanic Americans (HA).** Manhattan plot for (A) smoking initiation, (B) smoking cessation, (C) AUD, and (D) AC. The nearest genes for genome-wide significant (GWS) loci are shown.

Smkinit: smoking initiation; Smkcess: smoking cessation; AUD: alcohol use disorder; AC: alcohol consumption.


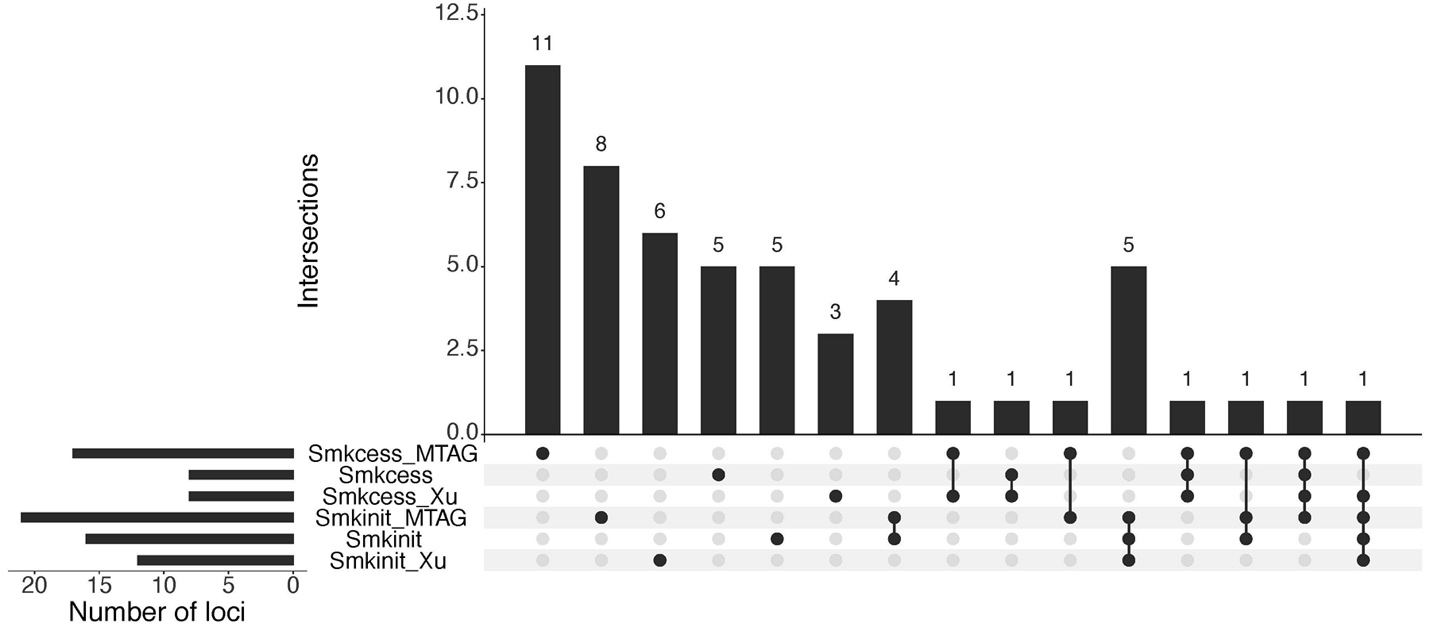


**Supplementary Figure 6: Upset plot for the number of genome-wide significant (GWS) loci identified by Multi-trait analysis of GWASs (MTAG) with alcohol use disorder (AUD), single-trait genome-wide association study (GWAS) and previous Million Veteran Program (MVP) GWAS.**

Smkinit: smoking initiation; Smkcess: smoking cessation; Smkinit_Xu/Smkcess_Xu: previous MVP GWAS on smoking traits reported by Xu et al.


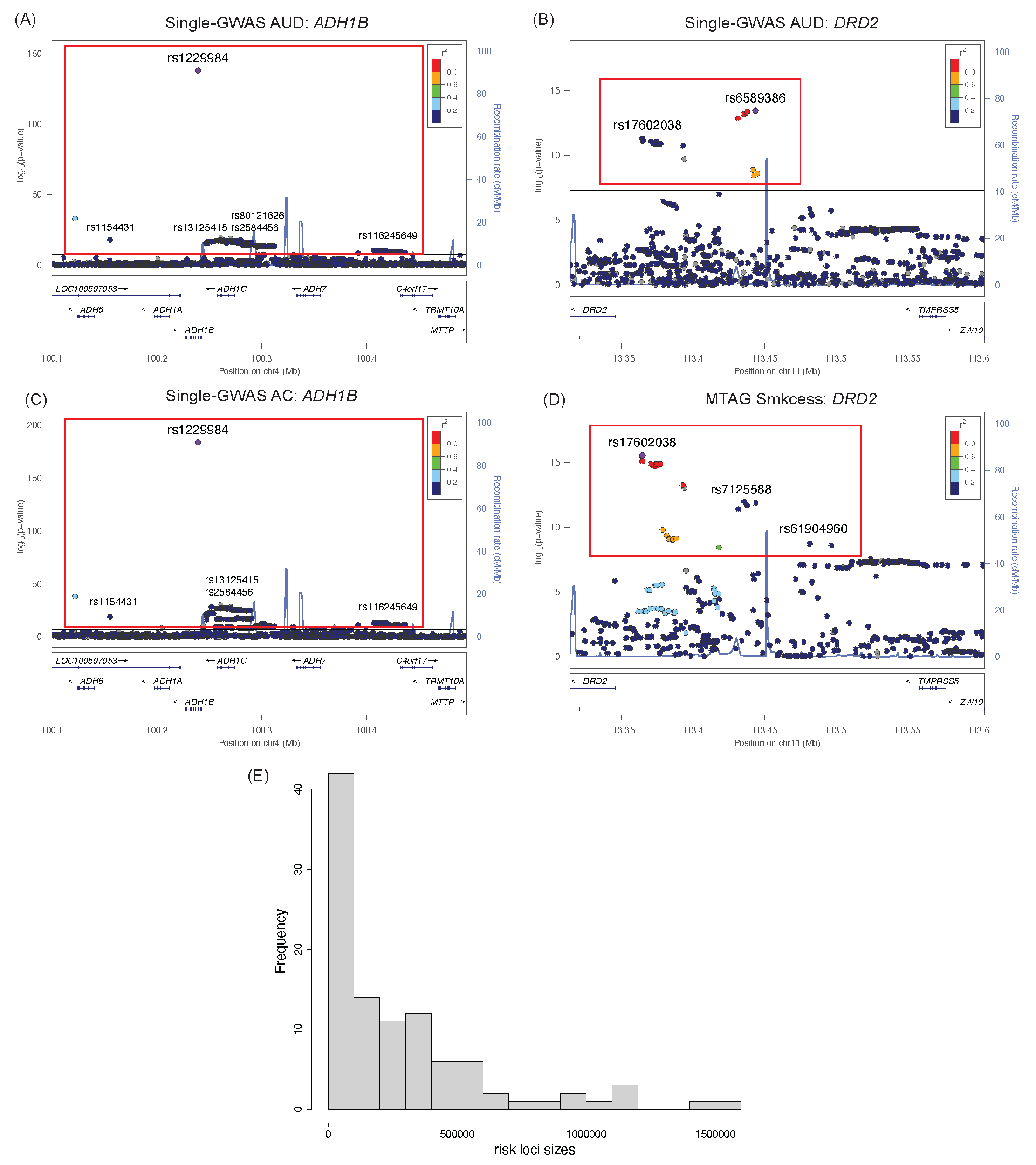


**Supplementary Figure 7: Merging of independent genome-wide significant (GWS) loci within 250kb and distribution of sizes for final loci.** We merged loci within 250 kb into a single risk locus for (A) AUD single-trait genome-wide association study (GWAS) at *ADH1B* locus, (B) AUD single-trait GWAS at *DRD2* locus, (C) AC single-trait GWAS at *ADH1B* locus, (D) smoking cessation multi-trait analysis of GWASs (MTAG) at *DRD2* locus. Nearby (within 250kb) independent SNPs (LD, r^2^ < 0.1) are marked, and merged into one single locus (red square). The final lead SNP for the merged locus (the top SNP with the minimum p value) was indicated in purple. (E) Histogram showing the distribution of risk locus sizes across all GWAS and MTAG.

Smkcess: smoking cessation; AUD: alcohol use disorder; AC: alcohol consumption.


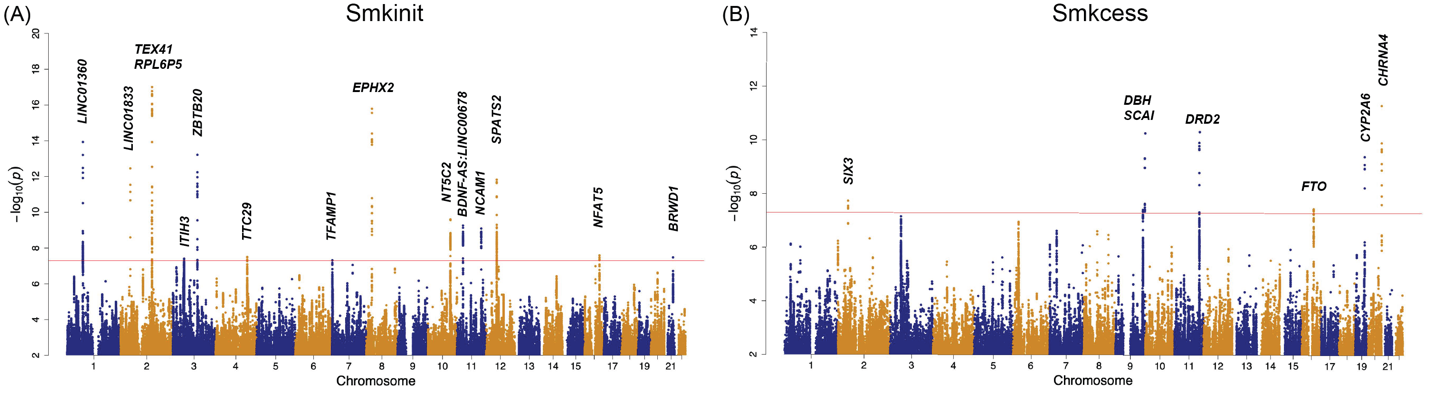


**Supplementary Figure 8: Multi-trait analysis of GWASs (MTAG) on two smoking phenotypes with alcohol consumption (AC).** Manhattan plot of MTAG with AC for (A) smoking initiation and (B) smoking cessation. The nearest genes to GWS loci are shown.

Smkinit: smoking initiation; Smkcess: smoking cessation; AUD: alcohol use disorder; AC: alcohol consumption.


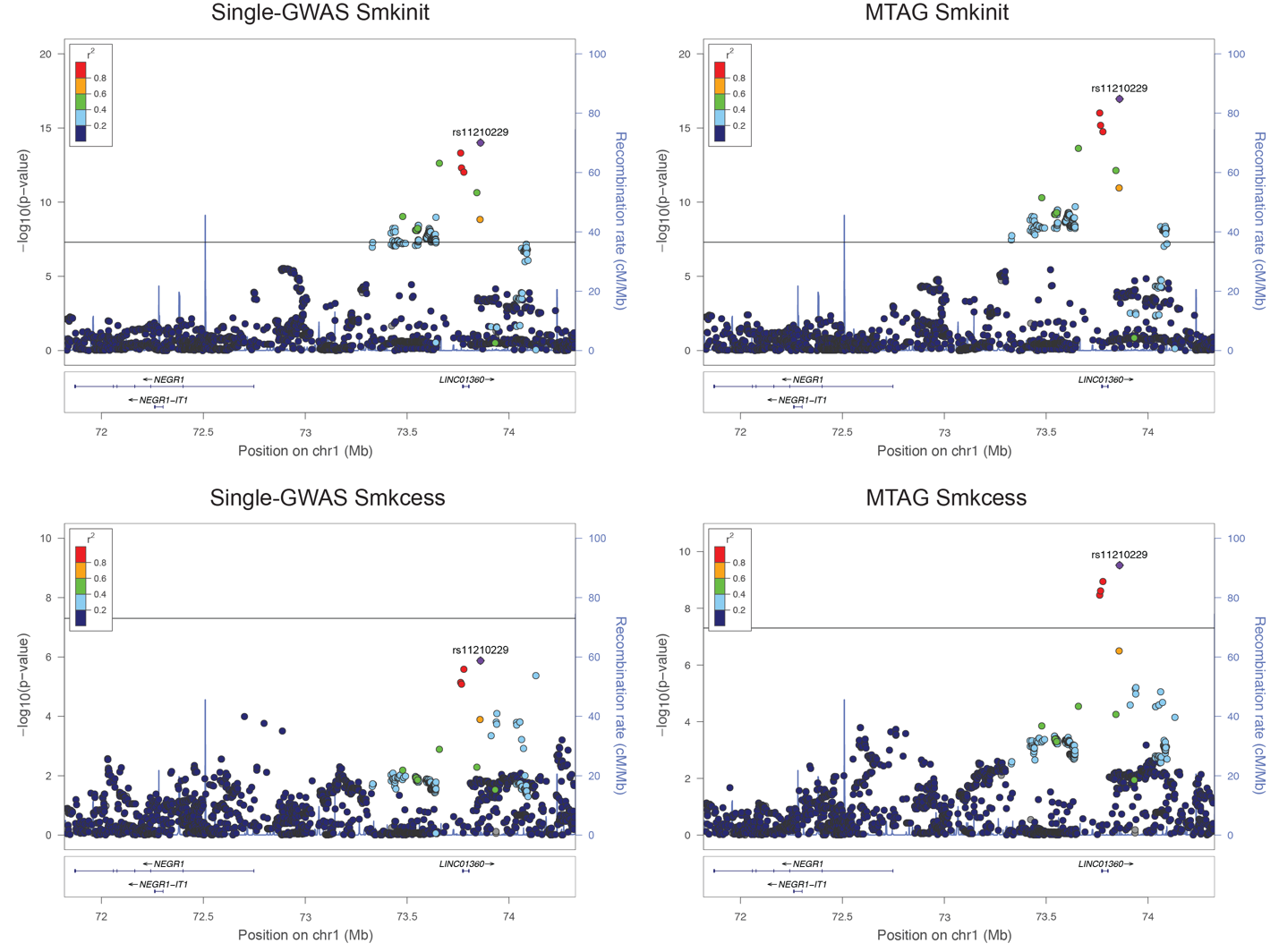


**Supplementary Figure 9: Stacked association plots of** **rs11210229 (*LINC01360*) in the 2 smoking-related GWASs.** The genetic variant rs11210229 mapped on *LINC01360* was identified as a colocalized SNP between AUD and 2 smoking related traits.

Smkinit: smoking initiation; Smkcess: smoking cessation; AUD: alcohol use disorder.
